# Women and health workers’ conceptualisations of reproductive coercion and abuse: a comparative synthesis from Brazil, Nepal, Palestine, and Sri Lanka

**DOI:** 10.1186/s12978-025-02112-z

**Published:** 2025-10-21

**Authors:** Manuela Colombini, Amera Shaheen, Poonam Rishal, Prabhash Siriwardhana, Claudia Garcia-Moreno, Loraine J. Bacchus, Sophie Morse, Erin Hartman, Ana Flavia d’ Oliveira

**Affiliations:** 1 Department of Global Health and Development, London School of Hygiene and Tropical Medicine, London, UK; 2 Faculty of Medicine and Health Sciences, An-Najah National University, Nablus, Palestine; 3 School of Medical Sciences, Kathmandu University, Kathmandu, Nepal; 4 Department of Social Sciences, Rajarata University of Sri Lanka, Mihintale, Sri Lanka; 5 Faculty of Medicine, University of Peradeniya, Peradeniya, Sri Lanka; 6 Department of Sexual and Reproductive Health and Research, World Health Organisation, Geneva, Switzerland; 7 Philip R. Lee Institute for Health Policy Studies, University of California San Francisco, San Francisco, USA; 8 Department of Preventive Medicine, School of Medicine, University of São Paulo, Sao Paulo, Brazil

**Keywords:** Reproductive coercion and abuse (RCA), Violence against women, Low- and middle-income countries (LMIC)

## Abstract

**Background:**

Reproductive coercion and abuse (RCA) is a hidden form of violence against women, involving controlling behaviours by a partner or family member to manipulate a woman’s reproductive autonomy, either to prevent or promote pregnancy. It correlates with partner violence, unintended pregnancy, contraceptive non-adherence, and poor sexual and reproductive health. However, research often oversimplifies RCA, treating it as a uniform phenomenon and neglecting its diverse manifestations. Additionally, there is scarce evidence on RCA in low- and middle-income countries. This study examines the conceptualisation and discourse surrounding reproductive coercion among health workers and women victims/survivors in Brazil, Nepal, occupied Palestinian Territories, and Sri Lanka. The main objectives include: 1) Investigating acts of reproductive coercion reported by women and health workers. 2) Exploring how health workers approach reproductive coercion in their practice. 3) Understanding the structural, institutional, and social barriers affecting victim/survivors encounters with reproductive coercion.

**Methods:**

We conducted 62 qualitative interviews with health workers and domestic violence victims/survivors across the four countries, plus three focus groups with women in Nepal. Data were analysed thematically.

**Results:**

The findings reveal that reproductive coercion emerged as a form of domestic violence across all settings studied. Reported acts of coercion and violence by both women and health workers included attempts to force pregnancy against a woman's wishes and to hinder contraceptive use, driven by jealousy or the desire to promote pregnancy. Perpetrators, mainly husbands and family members (particularly in-laws in Nepal and Sri Lanka), employed various coercive behaviours such as pressure, decision-making control, threats (e.g., leaving the partner or violence), verbal harassment, and physical violence. The analysis also underscored broader structural and social challenges constraining women's reproductive choices and health workers' responses, encompassing religious beliefs surrounding contraception and abortion, cultural norms regarding son preference (notably in Nepal), and restrictive health policies concerning abortion and spousal consent for family planning (observed in Nepal and Sri Lanka).

**Conclusions:**

The study emphasises the necessity for further research to comprehensively understand acts of reproductive coercion and abuse and guide health workers in effectively addressing this issue.

## Abstract (plain language summary)

Reproductive coercion and abuse (RCA) is a hidden form of violence where someone controls a woman’s choices about having children, either by forcing her to become pregnant or preventing her from doing so. This can have serious consequences, like unintended pregnancies and poor reproductive health. However, RCA is often misunderstood and oversimplified in research, particularly in low and middle-income countries (LMICs), where the issue has not been studied enough. This study looks at how RCA is understood by health workers and women in four countries: Brazil, Nepal, occupied Palestinian Territories (oPT), and Sri Lanka. The study aims to identify the types of reproductive coercion experienced by women, understand how health workers deal with it, and explore the cultural, social, and structural barriers that make RCA difficult to address. Researchers conducted interviews with 62 health workers and victims/survivors of domestic violence and held group discussions with women in Nepal. The findings show that RCA is a form of domestic violence, with women being pressured by partners and family members, especially in-laws, to either become pregnant or stop using contraception. Women in Brazil reported facing pressure (sometimes described during clinical encounters) not to seek abortions. Cultural beliefs like a preference for sons, religious views on contraception, and strict health policies added to the difficulties women face in controlling their reproductive choices. The study concludes that RCA is a complex issue shaped by cultural, religious, and social factors. It calls for more research to help health workers better understand and address reproductive coercion, especially in LMICs.

## Introduction

Reproductive coercion and abuse (RCA) is a significant public health issue [1–3], disproportionately affecting younger women [4, 5]. RCA prevalence varies significantly. A recent study estimating RCA rates across ten sites in Africa and Asia, reported a range from 20.3% in the Democratic Republic of Congo to 3.1% in Niger, highlighting notable geographical differences [6]. It involves behaviours that interfere with a woman's autonomy in making reproductive decisions, including contraceptive use and pregnancy choices [7]. Coercive actions, often accompanied by physical violence, range from promoting pregnancy (e.g. sexual violence, contraception sabotage, or pressuring a woman to continue a pregnancy) to preventing pregnancy (e.g., forcing abortion or contraception use) [7, 8]. Control, fear, and intent characterise these RCA actions [8]. Reproductive coercion may result from macro health policies (e.g. restrictive laws, lack of offer of information or access to contraceptive methods, selective offer of highly effective methods) [9] or from interpersonal relationships, which is the focus of this paper.

RCA is primarily perpetrated against women and young girls, mostly by their husband or partner [7, 10]. Though in some contexts, other family members, such as mothers-in-law, may also engage in it [11–13]. RCA can occur independently, as a distinct form of violence against women, though it is often viewed as the mechanism connecting intimate partner violence (IPV) to negative reproductive health outcomes, highlighting their close relationship [7, 14]. Perpetrators of IPV often attempt to control women’s fertility, significantly impacting their ability to negotiate safe sex, access and utilise contraception, and obtain family planning (FP) services. For example, an intervention study conducted with expectant couples and current parents in Rwanda revealed that women who experienced reproductive coercion in the past year were approximately 2.5 to 4 times more likely to report experiencing IPV [15].

Perceived motives for RCA behaviours are diverse and include son preference [16, 17], control over women’s lives and decision-making [16–18], rigid gender roles [16], family pressure [16], family formation or dissolution [17], entrapment and self-interest [18], and a desire for domination and control [18].

Beyond its impact on women’s reproductive autonomy, RCA is also associated with a range of adverse health consequences that are detrimental to the reproductive health and wellbeing of women and girls. These include increased risk for sexually transmitted infections (STI) [5], poor mental health [19], post-traumatic stress disorder [7], repeat use of emergency contraceptives, unintended pregnancies [1], and lower contraceptive self-efficacy [20]. Recent qualitative research has also shed light on its complex impact on women’s experiences of mothering, with some women feeling detached, resentful, and guilty toward their children [21]. Furthermore, RCA hinders broader achievements towards gender equality, economic prosperity, and sustainability [22–25].

### Reproductive coercion and abuse in the 4 study settings

This study is a sub-study of the HERA (HEalthcare Responding to violence and Abuse) project, which was implemented across four study countries—Nepal, Brazil, Sri Lanka, and occupied Palestinian Territory (oPT). Reproductive coercion and abuse, is a pervasive issue across the four countries, shaped by patriarchal norms, societal expectations, and cultural practices, with varying degrees of family or societal pressure influencing its prevalence, yet research and policy addressing the issue remain limited in all contexts.

In Nepal and oPT, RCA is deeply rooted in patriarchal systems, with strong family pressures—particularly from in-laws—driving women's lack of autonomy over reproductive decisions [26, 27]. Social norms, a preference for sons, and harmful traditional practices contribute to the prevalence of RCA [27–29]. Structural barriers also play a key role in oPT [30]. In both countries, abortion is legally restricted, but permitted under certain conditions [31, 32]. While health guidelines provide access to family planning services, they do not directly address RCA, and husbands often play a central role in contraceptive decisions [31].

In Brazil, RCA is less commonly studied, though male partners refusing to use condoms or suspecting women's infidelity if she asks for condom use is a noted form of coercion [33]. Unlike Nepal or oPT, societal pressure to have children has significantly declined, particularly in urban areas. Brazilian health policies prioritize access to family planning but do not explicitly address issues of coercion. Although abortion remains heavily restricted, recent legal changes have relaxed some reproductive limitations [34], such as the removal of the requirement for partner consent for sterilization. Additionally, Brazil's domestic violence law classifies preventing or forcing the cessation of contraception use as a form of sexual violence [35].

Sri Lanka is characterised by a high number of unwanted pregnancies [36, 37], societal expectations [38] and restrictive abortion laws [39] shaping women’s reproductive choices, with limited attention given to coercion in both policy and research. Sri Lankan health guidelines [40] lack a comprehensive definition of reproductive coercion though they stress the importance of providing women with information and options regarding contraceptive methods.

### Gap in research

Despite growing global evidence documenting the pervasiveness of reproductive coercion among women and girls seeking family planning services [10, 11], it remains overlooked in health interventions and research [8, 41]. Global guidance on how family planning health workers can identify and respond to RC is relatively new [42].

Most health interventions targeting RCA – though limited—have been implemented in FP clinics. The most well-known intervention to address reproductive coercion, ARCHES (Addressing Reproductive Coercion in Health Settings), was initially developed in the United States and is now being adapted for other LMIC settings [17, 43]. ARCHES is a brief, clinician-delivered universal education and counselling intervention in FP clinics aimed at reducing IPV, reproductive coercion and unintended pregnancies, whose effectiveness has been assessed in a randomised controlled trial [44]. However, effective models for addressing RC and IPV within other clinical services in LMICs have yet to be identified.

Furthermore, although health workers are essential in identifying and responding to RCA [45], there remains a significant research gap in understanding how these health workers and other non-health professionals address RCA. Most existing studies originate from the US [46], Canada [47] and Australia [48]. Additionally, contextual differences in how healthcare workers perceive and respond to coercive acts related to RCA also remain underexplored.

This paper aims to explore how RCA is conceptualised and understood by health workers, as well as by women who have experienced intimate partner violence, and that are accessing health services in four countries: Brazil, Nepal, Sri Lanka and oPT. We endeavour to answer the following research questions:

- What forms of RCA are experienced by women?
- What are health workers’ views of RCA?
- How do healthcare workers address RCA?

## Methods

### Study settings

The four HERA study countries were selected based on previous and ongoing research collaboration on violence against women. A detailed description of their national health systems contexts is provided elsewhere [49].

Study sites differed by geographical location and facility type. In Brazil, oPT, and Sri Lanka, the research involved urban public health facilities, including primary health care clinics in São Paulo, Brazil; Nablus, Jenin and Jerusalem in oPT, and hospitals in Colombo, Sri Lanka, In Nepal, the study was primarily conducted in rural, hospital-affiliated Outreach Centres (ORCs). Sri Lanka and Nepal also used One Stop Crisis Centres for referrals [49]. These One Stop Crisis Centres are crisis management facilities for survivors/victims of violence, providing integrated health, legal, and psychosocial support,

### Participant’s sampling, study tools and data collection methods

A purposive sample of 62 qualitative interviews, and 3 focus group discussions (FGDs), which discussed perceptions and experiences of RCA, was considered (see Table 1 for details about study participants). Health workers were purposively selected based on their experience providing care to women affected by IPV, while victims/survivors of IPV were identified through existing services at study sites. Female community volunteers were purposively selected for FGDs, based on their willingness and ability to reflect on community experiences with IPV. The Nepali research team considered group discussions appropriate and acceptable for exploring shared experiences of violence (including reproductive coercion) among women in that context. The number of FGDs was determined pragmatically based on feasibility, ethical considerations, and reaching data saturation.

**Table 1:** Type and number of respondents

	**Brazil**		**Nepal**		**Sri Lanka**	**oPT**
**In-depth interviews:**						
Health workers	13	7		13		22
Women	/	3		2		2
**Focus group discussions:**						
Female community health volunteers	/	3		/		/

Interviews were conducted between June 2019 and August 2020 and between November 2019 to February 2022. Data collection in some sites overlapped with the COVID-19 pandemic (which may have affected participant access and disclosure). All interviews and FGDs were conducted in local languages by trained researchers and took place at the study clinics (Brazil, Nepal, oPT, Sri Lanka) or a private place selected by participants (Brazil, Nepal). Research teams used study distress protocols and debriefed regularly to support their emotional wellbeing during interviews with victims/survivors of IPV. Interviews were audio-recorded and subsequently transcribed, and relevant sections from 15 transcripts were translated into English to facilitate the analysis across countries.

### Data analysis

Data was analysed thematically [50]. Each country team conducted their data analysis independently, starting with the co-development of a joint code frame used consistently across all four countries. Key themes were discussed during regular online workshops involving UK and local researchers. A summary matrix was created for cross-country analysis. Additionally, each country provided a detailed summary of their main findings, including English translations of illustrative quotes for each identified theme and sub-theme. The research team then discussed and compared the combined results from all countries during two virtual workshops. Reports were generated in Word tables to explore data across countries and themes. Finally, the main findings across countries were synthesised into key findings, jointly selected by the research groups.

### Ethical considerations and approvals

We adhered to the ethical and safety guidelines for conducting violence-related research set by the World Health Organization [51] to protect both study participants and researchers. Participants were informed about local psychosocial support services in case they required additional assistance. Ethical approvals for this study were received from the authors’ institutions.

## Results

We present the data according to three broad themes: i) perceived manifestations of RCA; ii) health workers’ views and awareness of RCA; iii) health workers’ responses to RCA. Table 2 summarises the key findings across the 4 study countries. While some RCA perceptions and responses were unique to specific contexts, several commonalities emerged across the four countries. These included the normalisation of male control over family planning, limited recognition of RCA as abuse, and lack of training.

**Table 2:** Summary of country-specific findings on manifestations of RCA, health workers’ awareness and responses to RCA

	**Nepal**	**Sri Lanka**	**Brazil**	**oPT**
Manifestations of RCA	The pressure to conceive male children was particularly strong, often resulting in severe emotional and physical abuse. In-laws in Nepal also exerted considerable influence over reproductive decisions, often overriding the woman's wishes	RCA often involved husbands controlling their wives'use of contraception, sometimes motivated by jealousy or suspicion of infidelity. Pressure to conceive more children was common, though not always recognised as coercive behaviour by healthcare workers and/or women	The control over reproductive choices was described not only as physical but also psychological, with husbands imposing religious beliefs to justify their opposition to contraception. Healthcare workers reported that women often hide their use of contraception from their husbands, fearing backlash	RCA often involved husbands or in-laws exerting pressure on women to have more children or to stop using FP methods. In some cases, RCA was linked to broader societal expectations regarding fertility and the role of women in maintaining family honour. Cultural norms heavily influence reproductive decisions, with in-laws and husbands often dictating the number of children a woman should have. There was also a noticeable emphasis on son preference
Health workers’ awareness and perception of RCA	Awareness of RCA was limited among health workers. Many did not explicitly label behaviours such as pressure from partners to discontinue family planning methods as reproductive coercion, seeing it more as a cultural or social issue rather than a form of abuse	Health workers showed limited awareness of RCA as a distinct issue. Choice of contraception was often viewed as a matter of couple decision-making, with the husband's consent playing a significant role	RCA was framed more broadly as an issue of limited community awareness rather than a form of violence that women experienced. RCA was often viewed in the context of a lack of education and resources regarding contraceptive methods	Awareness of RCA was somewhat higher among health workers, with some recognising it as part of intimate partner violence. Some workers acknowledged the role of cultural norms and the necessity of husband’s consent in FP decisions
Health workers’ responses to RCA	Health workers attempted to counsel women on the health risks of frequent pregnancies. However, they often felt helpless when women, under pressure from their families, chose to stop using contraception. Some health workers tried to engage husbands in discussions about use of FP methods but faced negative repercussions, including threats	Health workers attempted to advise women on the importance of using contraception and the health implications of repeated pregnancies. Some tried to discuss FP use with husbands, but cultural norms limited the effectiveness of these interventions. Compared to the other countries, there was a lack of systematic approaches to address RCA	Health workers focused on educating women about FP methods and their rights to make independent decisions about their reproductive health. Some health workers also engaged in community education efforts to increase awareness and reduce the stigma associated with contraceptive use	Health workers often counselled women about the health risks of not using FP methods, but faced challenges when dealing with family dynamics that prioritised male authority. Some health workers recognised the need for more training on how to handle RCA safely and effectively

Most women in the study were aged between 20–45 years, predominantly married, and with children. Health workers included nurses, doctors and GBV Focal Points (oPT only). FGDs participants included female community health volunteers (Nepal only).

###  Perceived manifestations of RCA experienced by women

**Fig. 1:**
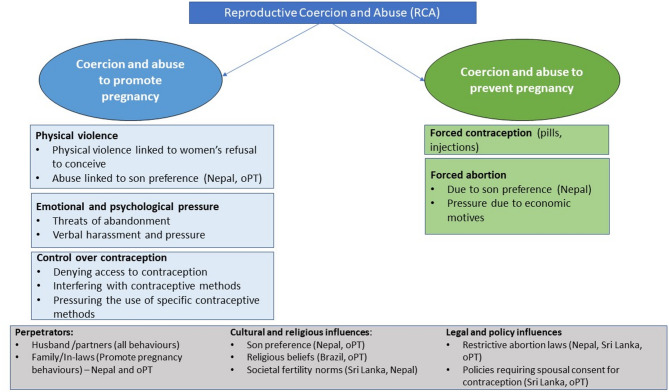
Summary of behavioural typologies of RCA

Two distinct categories of reproductive coercion and abuse emerged from our findings across different country contexts: coercive behaviours to promote pregnancy and coercive behaviours to prevent pregnancy (including to have or not to have an abortion when women wanted). These acts were primarily described by health workers, based on their observations in clinics and what women shared with them. Most were perpetrated by husbands, though in some instances, in-laws were involved as well. Behavioural typologies of RCA as reported by health workers (and some women) are summarised in Fig. 1 below.

#### Coercion and abuse to promote pregnancy

Pregnancy coercion was the most frequently reported type of reproductive coercion and abuse across all settings. This form involved various pressures exerted on women to become pregnant or to continue pregnancies against their will. Women reported emotional abuse, pressure, verbal threats, and physical abuse when they attempted to use contraception or expressed a desire not to conceive more children. A health worker from Brazil reported women feared abandonment and experienced threats of economic violence if they did not comply with their husbands’ fertility desires.

‘’We asked:'was the pregnancy planned?'She replied,"Well, I didn't want to, but my husband said that if I didn't get pregnant, he would leave me."[Health worker from Brazil]

Coercion related to controlling contraceptive use was another prevalent manifestation of RCA aimed at maintaining or forcing pregnancy. This involved actions such as denying women access to contraception, controlling decisions about contraceptive methods (regardless of any side effects experienced by the woman), and pressuring them to use or avoid longer acting contraception. Emotional abuse— expressed through pressure and controlling behaviours—was the most common form of coercion. Fear of retaliation led some women to conceal their contraceptive use, as reflected in the testimony of a woman in oPt, who hid her IUD use from her husband.

‘I: Are you using any contraceptives now ? R: Yes, an IUD. I: Does your husband know about it? R: Yes, at first he was against it. I got it without him knowing, and then I told him’. [Woman from oPT]

In oPt, a health worker described a situation where a woman’s husband refused to let her use contraception because he wanted more children, despite her having recently given birth and being physically weak.

‘There was a woman who came in after 4 months of childbirth, and 2 months pregnant because her husband doesn’t want her to take OCPs [oral contraceptive pills], she said: my husband doesn’t want that, and he wants me to keep having children” I wish you could see her body, it was frail, she couldn’t keep having kids’. [Health worker from oPT]

Across all countries, husbands were reported to exert significant control over decisions regarding contraception and fertility, frequently disregarding the woman’s health or preferences. However, the coercive nature of reproductive control—part of a broader pattern of abuse or violence—was often normalised as mere disagreement over family size.

‘Some women here say their husbands want more children, but they don’t. They don’t call it violence; it’s just how it is.’ [Health worker from Sri Lanka].

Extreme pressure to bear children was often rooted in cultural preferences for male children, as observed in Nepal and oPt, where son preference was a significant driver of reproductive coercion. Health workers reported instances where women were either threatened or physically beaten by their husbands, either for being suspected of carrying a female child or for refusing to conceive again soon after childbirth.

‘*If you give birth to a daughter, I will bury both the mother and daughter in a pit*’. [Health worker from Nepal].

‘And when I asked why she was beaten, she said she had two daughters. When she got pregnant again, she was told that it’s a girl’. [Health worker from Nepal]

‘She was told that he needed a son […] and she didn't want to bear a child then [after having recently aborted a female foetus]. And therefore, she was beaten; she had bruises all over her body'. [Health worker from Nepal]

Health workers’ narratives highlighted how social and cultural beliefs were deeply intertwined with reproductive coercion and abuse, —particularly regarding the control of women’s contraceptive use across all the studied regions. In Sri Lanka, FP use was often stigmatised and associated with extramarital relations, largely due to male jealousy and dominance. Both health workers and women reported that jealousy and suspicion frequently led husbands to forbid their wives from using contraception, fearing it might enable extramarital affairs.

‘Some men think that if a woman is using contraception, it’s because she wants to have an affair. They don't trust their wives, so they force them to stop using any method of family planning’. [Health worker from Sri Lanka]

‘This [forcing women not to use FP] is mostly attributed to […] jealousy […] We have met some married couples where one partner suspects the other spouse and therefore forces the other partner not to use family planning methods’. [Health worker from Sri Lanka]

One woman shared that her husband wanted her to be pregnant constantly, believing it would keep her busy with the children while he pursued extramarital affairs.

‘He always asked to make a baby. There were also economic issues. They didn’t even matter to him. He is a little bit. He had several other affairs with other women. That's why he wanted to have many children in order to keep me occupied at home all the time’. [Woman from Sri Lanka]

In Brazil and oPt, religious beliefs played a crucial role in preventing women from using contraception. Both Catholic and Islamic teachings were often cited as reasons behind male partners’ rejection of contraceptive use by their wives. A nurse in Brazil recounted the story of a woman who did not want to get pregnant but was pressured by her husband, who believed that contraception (‘the morning pill’) violated their religious practices. This pressure led her to experience postpartum depression after an unwanted pregnancy.

‘She no longer uses contraceptives precisely because of her partner, who thinks that they are Catholics. He said that contraception couldn’t be used, that God hadn’t given it to men. [..] He [husband] was kind of a religious practitioner, in which contraception is not seen in a good light, that contraception it's a kind of continuous abortion, he thought that.’ [Health worker from Brazil]

‘…her husband refuses [FP use] because he wants'the boy.'He is very religious and an Imam of a mosque. [..] He considered birth control “haram” [prohibited in Islam] [Health worker from oPT]".

Some respondents in Nepal also stated that religious and fatalistic views further contributed to RCA. An auxiliary health worker shared how some men believed that the number of children they had was determined by divine will, and any interference from health services was seen as unacceptable:

‘Her husband [aged 74] says: ‘[My wife] bears as many [children] as God gives.’ He doesn’t believe in using contraception because he thinks it’s against God’s will."[Health worker from Nepal]

Mother-in-law interference was also reported in some contexts, notably in Nepal and oPT, where women were pressured to have more children and often denied the right to use contraception. An example from oPt highlighted this, where a woman was forbidden by her mother-in-law from tying her tubes despite being physically exhausted from frequent pregnancies.

‘My mother-in-law… she loves kids. She always tells me to get pregnant and have more children.’ [Woman from oPT]

‘I had one case with another [woman] who was suffering from exhaustion. She has her own kids and lives in the family home […] and her mother-in-law is forbidding her from using any contraception or tying her tubes […]. She didn’t have any apparent physical signs of abuse, but they forbid her from getting her tubes tied. They’re telling her: “we want more kids but her body is tired”.’ [Health worker from oPT]

#### Coercion and abuse to prevent pregnancy

Conversely, in some situations, women were pressured – or forced – to use contraception (e.g. injection or tubal ligation) to prevent pregnancy by partners or in-laws. Coercive behaviours related to preventing pregnancy were reported by our respondents (primarily health workers), but were less common than coercion to promote pregnancies. These included forcing contraceptive use by husbands and in-laws. A Nepali woman recalled being coerced into taking contraceptive pills against her will, as her partner threatened to leave her if she refused.

‘He used to pressure. Every time you have to eat it [contraceptive pill]: “if you don’t eat then leave me”; now after marriage when your parents give you in hand to your husband then you cannot leave your life partner.’ [Woman from Nepal]

In oPt, a health worker recounted the story of a woman in a polygamous marriage who was forced to get an injection to limit her ability to get pregnant.

‘Once, there was a woman who wanted to get pregnant. She was the second wife, and she wanted to have a baby, but her husband didn’t want her to. The first wife, who was in control of the household, didn’t want her to get pregnant either. They would force her to come and get the contraceptive injection against her will to prevent her from getting pregnant.’ [Health worker from oPT]

Abortion coercion, though less commonly reported, was a significant issue in Nepal, where cultural practices favouring sons led women to abort female foetuses.

‘His father said he wants to have a son [but] when I delivered again I did not have that, it did not work out and then I had to do abortion, then I did an abortion’. [Woman from Nepal]

In one case, a woman experienced two abortions under different circumstances. The first abortion occurred due to financial difficulties, while the second happened after her husband insisted, despite her reluctance, using a false claim about the effects of contraceptives on the foetus.

‘I had also gotten pregnant after 9 months of marriage and got abortion twice; the first time I wanted to have the baby but he said that since we were both jobless and have nothing there is no use of having a baby now; to which I also agreed and the second time he told me to get an abortion because even after having the pill I got pregnant; so he said that the pill will make the baby bad; I was reluctant but believed him and had the abortion.’ [Woman from Nepal]

### Healthcare workers’ recognition and understanding of RCA behaviours

We distinguish here between health workers’ recognition of and understanding with RCA, and their actual responses in clinical settings. This separation allows us to highlight a key gap between recognition and action.

The extent to which health workers encountered RCA varied across the four countries. While healthcare workers spoke of women expressing pressure from their partners to conceive or discontinue FP methods to promote pregnancy, many health workers did not directly associate these situations with abuse. Instead, they thought that husbands’ influence on contraceptive use was part of couple-decision making.

‘Here, it’s common for the husband to have a say in whether his wife uses contraception. It’s seen as part of couple decision-making’. [Health worker from Sri Lanka]

Perceptions of RCA varied noticeably across countries, often shaped by local cultural norms and policy context. In Sri Lanka and oPT, for example, family planning was often viewed as a decision requiring the husband’s consent (even though there was no mandatory requirement) and health workers felt women were unable to act independently in these matters and required it for fear of family relation (oPT). Such statement reflected deeply ingrained patriarchal attitudes and gender biases, which persisted even when not supported by legal or clinical policy requirements.

‘Even if a woman wants to use family planning, she can’t go against her husband’s wishes.’ [Health worker from Sri Lanka]

In contrast, some health workers seemed to have a broader understanding of RCA, often framing it as an issue of limited community awareness rather than a form of violence. For instance, a Brazilian health worker remarked that “*If women had more knowledge about FP methods, they wouldn’t face these issues. It’s not so much coercion but a lack of education*.” This contrasted with some health workers (in Brazil and oPt), who had a stronger belief that getting a woman pregnant without her consent—even in the absence of active interference or violence from a husband—constituted violence and that the decision should be the woman’s alone.

‘This [denial of contraception] is an oppression of women. It’s her body, it’s supposed to be her decision. No one should be able to impose [their decisions] on her or forbid her from tying her tubes, using contraception… we’re trying to convince as many women as we can reach, that it’s your body and no one else should have control over it or force you into anything.’ [Health worker from oPT]

‘Just because you are being forced to do something. No matter how much this person, suddenly the woman in this situation, wants to, says: “Ok, I'm going to get pregnant. Let's have this child, I don't want to have it, but you do, so let's have the child”. Even if the sexual relations are consensual, even if she is having a consensual relationship, and she in fact wants to have these sexual relations, the outcome, which is to have the child that she does not want to have this child, that’s the violence bit.’ [Heath worker from Brazil]

However, even within oPt, some health workers emphasised the necessity of husband’s consent for contraceptive use, reflecting internal cultural variation and/or health policies.

### Healthcare workers’ responses to RCA

The responses of Health workers to manifestations of RCA varied, often reflecting their limited awareness and understanding of the issue. Their actions ranged from expressing confusion about why women would not use contraception when they did not desire more children, to counselling husbands on contraception use.

Health workers’ narratives about women’s perspectives of RCA revealed that women often did not identify coercive behaviours as such. Instead, they spoke about hiding their contraceptive use due to fear of their husbands’ retaliation. Consequently, some healthcare chose not to inquire further about coercive behaviours.

‘Women come to us hiding their family planning use, but they don’t describe it as coercion. It’s more about fear of their husbands finding out.’ [Health worker from Nepal].

A Brazilian health worker found it surprising that a woman chose not to use contraception due to her husband’s influence, despite her desire to avoid pregnancy. However, he failed to recognise the underlying power dynamics involved. In contrast, a health worker from oPt reflected on his own behaviour, acknowledging he should have asked more probing questions.

“It seemed odd to me, but I didn’t think to ask more. Maybe I should have.” [Health worker from oPT]

“And I thought it was strange that she adhered to, you know, not using contraception and then she got pregnant. Something that she didn't want, she said she didn't want to be a mother and she ended up getting pregnant.’ [Health workers from Brazil]

Though some health workers in oPt and Brazil questioned women’s decisions to forgo contraception under their partners’ influence, several across the countries expressed efforts to counsel women on the importance of using FP and the negative impacts of repeated pregnancies. However, some health workers found it challenging when women lacked decision-making agency, or felt helpless when women decided to discontinue FP despite their advice.

‘I try to advise women about the health risks of having too many children too quickly, but it’s difficult if the husband is against it.’ [Health worker from oPT]

‘I knew she didn’t want more children, but she said her husband wouldn’t allow it. What could I do?’ [Health worker from Nepal]

A female community health volunteer advised a woman to use Depo (a both control injection) as a solution to her difficulty in using contraception without her husband's knowledge.

‘They [women] say that when you get the implant, their husbands might find out. When you use an IUD, it might prick during intercourse, and that’s when they notice. I already spoke to one of the Bainis (younger sisters, used here as a respectful term) who didn't tell her husband and got an IUD. When they had intercourse, he felt it, got angry, and she ended up removing it. They later had a son. The Didi-Bainis (older and younger sisters, also used respectfully) have these kinds of problems. When one woman had trouble with the implant [because she was using it covertly for fear of husband knowing as he got angry before], I advised her to try Depo. I told her: “If you take Depo, he won’t know. It might hurt for a day, but you can manage it yourself”.'[FGD participant from Nepal]

In some cases, health workers attempted to engage with male partners directly to advocate for delaying pregnancies or allowing their wives to use contraception. One health worker shared an experience where a woman’s husband refused contraception because he wanted a son. She explained the focus was not on preventing pregnancy, but on delaying it until the woman regained her health.

‘Her husband of course refused because he wants ‘the boy’ […]. I told her we don’t want to prevent pregnancy, we just want to delay it until you get back your good health. She was convinced, and he (the husband) promised to give her a rest period [from getting pregnant].’ [Health worker from oPT]

In other situations, a health worker in Brazil addressed the need for separate consultations with both partners, acknowledging that in some cases, men may not recognize their behaviour as abusive or coercive. These efforts aimed to educate and shift harmful cultural perceptions around marital roles, sexual rights, and reproductive decisions.

‘Depending on the type of violence that makes sense this separate evaluation. To invite her to an appointment alone, to invite him to an appointment alone too. Because there is another aspect to it. I don't know if it's bad to talk about it, but culturally speaking, sometimes there’s a man who's subjecting his wife to this situation of violence and doesn't even realise it. And he doesn't even realise it's violence...It also happens that a woman thinks that it is her duty to have sexual intercourse at home, even if she doesn't want to. Sometimes there's a man who thinks the same way...[So it is time] to work on it, to see what his point of view is like about what's happening in the relationship. I think it's worth it, always, at least one appointment of each one separately and one appointment together. Because there are things that we will be able to talk about together...’. [Health worker from Brazil]

However, these approaches involving male partners were often fraught with challenges including safety concerns. One health worker in Nepal recounted a negative experience where discussing contraception use with a husband led to threats, underscoring the risks health workers face when addressing reproductive coercion and abuse.

‘I tried talking to the husband, but he got angry and threatened me. It made me realise we’re not trained to handle these situations safely.’ [Health worker from Nepal]

Organisational issues within the healthcare systems also influenced health workers’ responses in some settings. Some nurses referred women who reported experiencing RCA—primarily being forced to forgo contraception—to doctors for further guidance on family planning, though this was not always effective. For instance, a hospital nurse in Sri Lanka described difficulties in consulting a doctor for FP advice due to delays, which further complicated efforts to support women facing family pressure to discontinue contraception.

‘It’s hard to get through to the doctor for advice on family planning. The process is slow, and by the time we can do something, the woman might have already faced pressure from her family to stop using contraception’. [Health worker from Sri Lanka]

Gaps in training related to identification and response to manifestations of RCA was a recurring theme across all interviews with health workers. There was a general sense of confusion and lack of knowledge and training on how to effectively identify and address coercive behaviours related to pregnancy promotion and prevention.

## Discussion

To our knowledge, this is the only comparative study on health workers’ experiences and responses to reproductive coercion and abuse across 4 LMIC countries. Our findings show that RCA is a significant problem across the four countries, driven by cultural, religious, and social norms, and perpetrated by husbands, with in-laws playing a role, especially in Nepal and oPT. RCA took two forms: coercion to either promote (pregnancy coercion) or prevent pregnancy, often involving verbal, emotional, or physical abuse when women resist. In Nepal and oPT, son preference fueled RCA, while in Brazil and oPT, religious beliefs (Catholicism and Islam) opposed contraception. In Sri Lanka, jealousy and suspicions of infidelity led husbands to control contraception to ensure their wives’ fidelity.

Our results highlight the complex and context-specific nature of reproductive coercion and abuse, demonstrating that while forms of abuse may be similar, the underlying motivations and justifications vary significantly by cultural, religious, and social factors. We found that cultural drivers like son preference, religious and community norms, gender power imbalances, and organisational practices (such as attitudes and health policies) condone RCA and hinder women’s reproductive choices. These drivers have been similarly discussed in the literature [25, 52]. Understanding the acts and perpetrators of reproductive coercion is essential for shaping services that support women and health workers, as well as for reducing gender-based discrimination in countries where son preference is still prevalent [53].

We provide insights into healthcare workers'awareness of reproductive coercion, how these perceptions vary across settings and how they align with global research. In our findings, RCA was interpreted differently depending on the cultural context: in Sri Lanka and Nepal, it was seen as part of couple decision-making, while in Brazil it was considered a community education issue, with health workers attributing RCA to a lack of contraceptive knowledge. In oPt and Brazil, RCA was also more explicitly recognised as a form of oppression of women, even in the absence of a physically violent partner. In Brazil, in particular, some reproductive health policies undermine a woman’s autonomy over her body, thereby perpetuating a different but no less harmful form of oppression [54]. As pointed out in our findings, the violation lies in forcing an unwanted pregnancy, whether or not the woman consents to the act of conception itself.

Despite these differences, one common theme across all countries was the lack of training and preparedness among healthcare workers to know how to respond, leaving many feeling unequipped to intervene effectively. RCA was rarely acknowledged as a form of abuse, with health workers often viewing it through the lens of cultural and societal expectations and policy requirements that gave male authority dominance in family planning decisions. Therefore, it is critical to also consider health policy contexts that reinforce gender inequalities, such as requirements for husband’s authorisation for certain reproductive health services, including contraception and abortion. These policy constraints, which perpetuate social norms, can indirectly coerce women and restrict their reproductive rights. It is important to distinguish between violent/abusive acts by partners or in-laws to interfere with contraception, as seen in the context of intimate partner violence, and coercive social norms or policies. While both restrict women’s reproductive autonomy, they require different approaches.

These findings align with existing literature from high-income countries, where healthcare workers are also reported to struggle with recognising and addressing reproductive coercion. Recent studies by Levesque [47] and Tarzia [18] emphasise the importance of health provider interventions like listening, identifying coercive signs, discussing relational contexts, and creating a safe environment for disclosure. However, even in these countries, challenges such as lack of time, inappropriate settings, and inadequate training hinder effective intervention. Our study adds nuance by showing how these challenges are further compounded in LMICs by deeply entrenched gender norms and power imbalances, making it even harder to address reproductive coercive behaviours.

The literature on partner violence and reproductive coercion points to promising intervention strategies that adopt human rights and women centred approaches, such as ensuring private consultations with women, focusing on confidentiality, respecting autonomous choice, and building a supportive environment for disclosure [48, 55, 56]. These practices are particularly relevant to our findings. While reproductive coercion is often under-recognised globally, our research highlights the added complexity of addressing it in LMICs, where healthcare workers must navigate not only medical concerns but also policies, cultural and social norms that discriminate against women and perpetuate male control over reproductive decisions. Tailoring intervention strategies to these unique contexts is essential to empower healthcare workers and support women in regaining control over their reproductive health and rights. Healthcare systems should prioritise training and supporting healthcare workers to recognise signs and respond to RCA, but also to educate women, discuss and offer strategies and resources to make informed reproductive choices.

Organisational practices that involve men in family planning decision-making are important. However, mediation with husbands should be approached with caution—as also recommended in the literature [55], as this may increase safety risks to both women and health workers. Discreet contraceptive options, such as injectables, can help women navigate patriarchal control over their reproductive health. More research is needed to explore how healthcare workers can better identify and support women facing RCA, and to develop strategies beyond family planning settings. Given the strong link between RCA and IPV [57], it is crucial that health workers in primary or maternal health are also trained to identify and respond to RCA.

Lastly, in settings like Nepal and oPT, where in-laws play a significant role in RCA, interventions must go beyond focusing solely on couples to address the broader family structure. Community-wide education that challenges male dominance in reproductive decision-making—and misinformation around contraception—is essential. Health systems, connected to communities through health workers and health promotion programs, can strengthen these efforts by fostering community-based models to prevent RCA and IPV.

### Limitations

One limitation of the study is the small number of women we interviewed about reproductive coercion (RCA), which restricted our insights into their experiences. While the perspectives of health workers offered valuable context regarding their encounters with women facing RCA, they were primarily based on what women shared or what health workers observed, sometimes lacking deeper context. Additionally, the women we interviewed were victims/survivors of partner abuse, highlighting the interconnection between RCA and intimate partner violence. Finally, as this study is a sub-study of an IPV intervention project, the focus on RCA was secondary, which may have limited the depth of our findings on this specific issue.

## Conclusion

The findings of this study underscore the cultural specificity of RCA, highlighting how patriarchal, religious, and social norms shape reproductive coercion across different contexts. While the core manifestations of RCA—whether coercion to promote or prevent pregnancy—are consistent, the motivations and justifications for these actions vary widely, making it crucial for healthcare workers to be culturally informed when addressing RCA.

Addressing these barriers requires structural changes within health systems, including more comprehensive training for health workers (both in-service and pre-service education), and interventions that prioritise women's reproductive autonomy over male or family control. In some contexts, carefully designed community-based awareness activities engaging husbands and mother in-laws may help challenge harmful norms and foster more supportive environments for reproductive autonomy. However, such efforts must avoid reinforcing male control and prioritise women's safety and agency. By doing so, health systems can help women assert their right to make autonomous decisions about their reproductive health, free from coercion and abuse. Further research should explore how healthcare workers in LMICs perceive RC, and which strategies to identify and respond to it are most effective across different settings.

## Data Availability

No datasets were generated or analysed during the current study.
